# Blood pressure in children with sickle cell disease is higher than in the general pediatric population

**DOI:** 10.1186/s12887-022-03584-9

**Published:** 2022-09-15

**Authors:** Juan C. Kupferman, Janet E. Rosenbaum, Marc B. Lande, Stella Stabouli, Yongsheng Wang, Daniella Forman, Dimitrios I. Zafeiriou, Steven G. Pavlakis

**Affiliations:** 1 Department of Pediatrics, Maimonides Medical Center, 977 48th street, Brooklyn, NY 11219 USA; 2 School of Public Health, SUNY Downstate Health Sciences University, Brooklyn, NY USA; 3 Department of Pediatrics, University of Rochester, Rochester, NY USA; 4 1st Department of Pediatrics, School of Medicine, Faculty of Health Sciences, Aristotle University of Thessaloniki, Thessaloniki, Greece; 5 College of Medicine, SUNY Downstate Health Sciences University, Brooklyn, NY USA

**Keywords:** Pediatric hematology/oncology, Sickle cell Anemia, Hypertension, Systolic, Childhood, Pediatrics

## Abstract

**Background:**

Sickle cell disease (SCD) is associated with an increased risk of cardiovascular disease that may be due to a variety of possible risk factors, including abnormal blood pressure. Blood pressure (BP) of children and adolescents with SCD has been reported to be lower compared to the BP of the general pediatric population.

**Methods:**

To confirm this prior observation, we compared reference BP values for children with SCD with reference BP values of the general pediatric population. We hypothesized that children with SCD do not have lower BPs than children without SCD.

**Results:**

Systolic BP differed for both males and females, over the different age groups between pediatric subjects with and without SCD. Systolic BP was higher in children with SCD, in both obese and non-obese populations. Diastolic BP did not differ between the groups.

**Conclusions:**

Our analysis demonstrated that systolic BP values are indeed higher in children with SCD than in the general pediatric population. This finding is consistent with the most recent literature showing abnormal BP patterns in the SCD pediatric population utilizing 24-hour BP monitoring devices. This is an important step for recognizing abnormal BP as a risk factor for cardio- and neurovascular events in SCD.

**Supplementary Information:**

The online version contains supplementary material available at 10.1186/s12887-022-03584-9.

## Background

Sickle cell disease (SCD) is associated with an increased risk of cardiovascular disease that may be due to a variety of possible risk factors, including hemolytic anemia and abnormal blood pressure [1–5]. Blood pressure (BP) of children and adolescents with SCD has been reported to be lower compared to the BP of healthy children without SCD [6, 7]. Based on this assumption, “relative” hypertension had been proposed as a risk factor for vaso-occlusive complications in SCD, including both overt and silent cerebral infarcts [6, 8, 9].

More recent studies have questioned the “low BP” hypothesis in SCD [10]. With the increased use of 24-hour ambulatory blood pressure monitoring (ABPM), children with SCD have been found to have abnormal BP patterns, including ambulatory hypertension, decreased nocturnal BP dipping, and masked hypertension [11–13]. Confirming whether children with SCD have lower reference BP values than children without SCD is an important step for defining BP abnormalities that are risk factors for cardiovascular events. Our analysis compared reference BP values of children with SCD with reference BP values of children without SCD. We hypothesized that children with SCD do not have lower BPs than children without SCD, contrary to current belief.

## Methods

We compared BP values for children with SCD with reference BP values for general population children without SCD. In this study, we did not evaluate determinants of BP.

### Data

Reference BP values for children without SCD were obtained from the most recent BP tables in the 2017 clinical practice guideline (CPG) for screening and Management of High Blood Pressure in children and adolescents (secondary data analysis, data publicly available) [14]. These normative data are based on the first BP measured during screening of 63,227 male and female children for each age and height. Like the older tables from the fourth report published in 2004, the new tables include systolic BP and diastolic BP values arranged by age, sex, and height percentile. However, in contrast to the fourth report, the new BP tables are based on normal-weight youth BP measurements from children and adolescents with body mass index (BMI) < 85th percentile, below the overweight cut-off. We also compared the SCD BP charts tables with the 2004 fourth report on BP that included children of all BMI percentiles as a robustness check [15].

For children with SCD, we used BP measurement data from the Silent Cerebral Infarct Multicenter Clinical Trial (SIT) [16, 17]. The SIT trial is a multicenter randomized controlled clinical trial that assessed whether blood transfusion can prevent recurrence of cerebral infarcts in children with prior silent infarcts. Elegibility included subjects 5 to 15 years of age with a confirmed diagnosis of HbSS or HbSβ°, with no history of focal neurologic deficits and no treatment with hydroxyurea or chronic transfusions [17]. Data available through 2012 were included from academic centers in North America (23 in USA, one in Canada) and Europe (4 in United Kingdom and 1 in France) Serial systolic and diastolic BPs were obtained from 944 and 943 pediatric subjects, respectively. Participants had a median of 5 BP measurements over a median follow-up of 3.3 years.

For this trial, quantile regression was used to generate estimates of the percentiles of height, weight, systolic and diastolic BP by age and gender, to construct the growth curves from the recorded data. More specifically, restricted cubic splines were used for the variable age to obtain smoothed curves, and cluster bootstrap methods were used to adjust the model-based standard errors for repeated measures. New tables that included the 50th, 90th and 95th percentiles of systolic and diastolic BPs for different height percentiles were constructed for males and females, based on these mathematically derived BPs [16]. Some children were on corticosteroids (8%), and 24% had asthma that could be associated with sleep disturbances at night and higher BP. We did not match the SIT trial data and CPG data by race.

Supplementary Table 1 presents BP measurements (50th, 90th and 95th percentiles) of children with SCD and of children without SCD (2004 Fourth report and 2017 CPG) for different heights (5th, 50th, 90th and 95th percentiles) [14–16].

### Statistical analysis

In this study, we assess whether children with SCD had higher BP than children without SCD. The BP data are matched by age, gender, and height percentile. For each combination of age, gender, and height percentile, we have diastolic and systolic readings for the SCD group, 2004 reference data, and 2017 reference data. Within each matched height percentile, we used the Wilcoxon signed-rank test to evaluate whether the distribution of BP differed between SCD and each set of reference children (2004 and 2017). Under the null hypothesis, on average the difference of systolic and diastolic BP between SCD and reference children for each combination of age, gender, and height percentile would be 0, which is tested by the Wilcoxon signed-rank test. We analyzed the data in R version 4.0.2 using a two-sided significance level of 0.05.

## Results

**Fig. 1:**
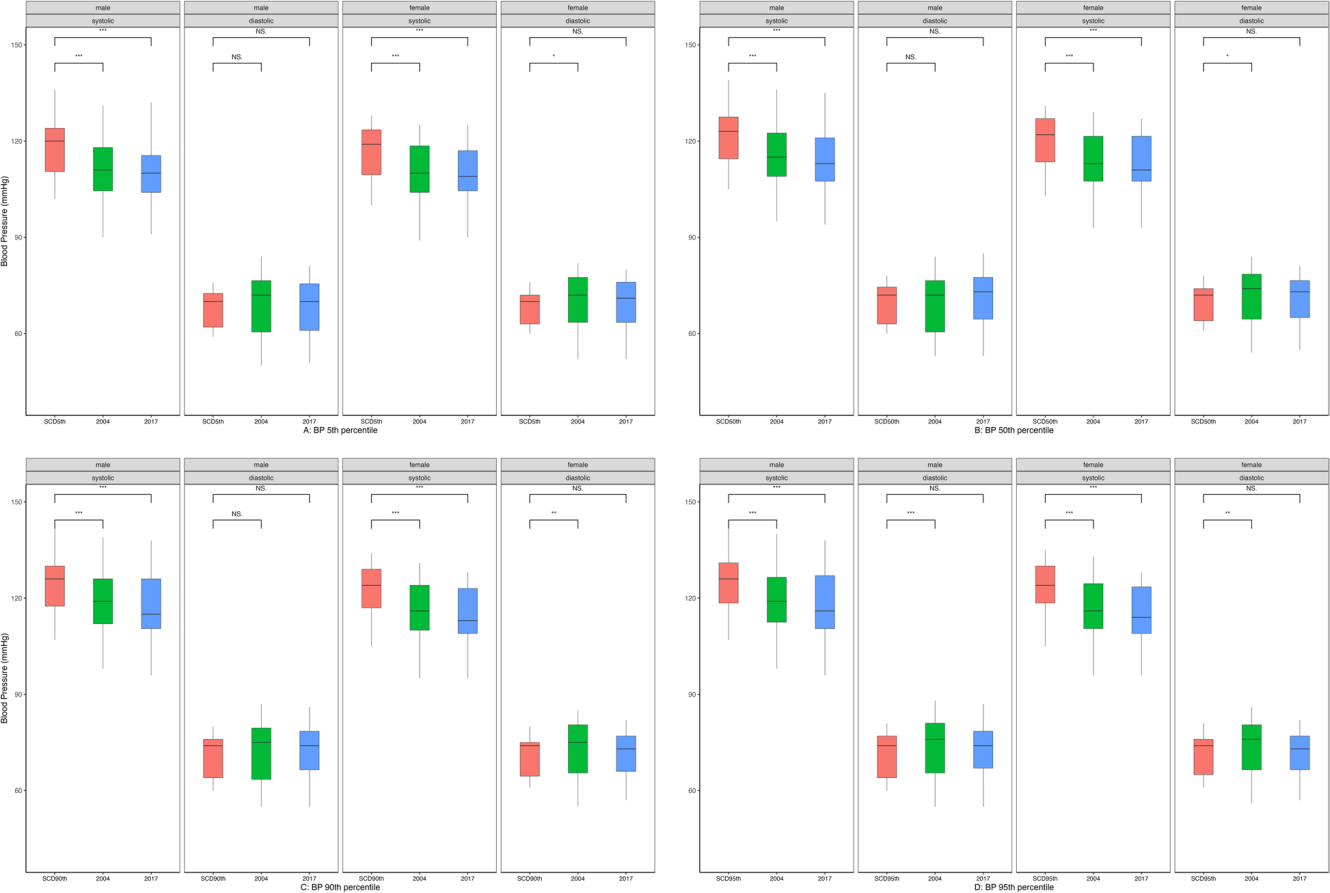
Comparison of blood pressure of children with and without sickle cell disease (SCD) by sex and height percentile. Significance codes: *** = *p*< 0.001, ** = *p*< 0.01, * = *p*<0.05, NS = not significant (*p*>0.05) Red = SCD, green = non-SCD 2004, blue = non-SCD 2017

Figure 1 compares BP of children with and without SCD by sex and height percentile. Systolic BP differed for both males and females, over the different age groups between pediatric subjects with and without SCD. Systolic BP was higher in children with SCD, in both obese and non-obese populations. Diastolic BP did not differ between the groups.

In addition to the differences found in BPs, height percentiles were lower in children with SCD in the SIT trial compared to children without SCD in the 2017 CPG [14, 16]. For example, the 95th percentile of height was 118.3 cm vs. 120.3 cm for a 5-year-old male and 163.8 cm vs. 173.4 cm at 13 years in the SCD and non-SCD groups, respectively. In girls, the 95th height percentile was 115.9 cm vs. 120 cm for a 5-year-old and 164.8 cm vs. 170 cm at 13 years, respectively.

## Discussion

Our analysis showed that BPs of children and adolescents with SCD are higher than of children without SCD, in contrast to prior studies. In addition, height percentiles were lower in children with SCD in the SIT trial compared to children without SCD in the 2017 CPG [14, 16]. Because height percentiles were lower in SCD than general populations, the BP elevation in these children is even higher: our results are likely biased away from the null of no effect because short children have lower BPs than tall children. These findings have important clinical implications for the interpretation of research studies as well in the decision to treat children with SCD and elevated BP.

Early studies reported lower BPs in children with SCD [6, 7]. Pegelow et al. compared 3317 subjects with SCD to those reported by NHANES I and II and found that BP was lower in SCD at median and 90th percentile. The BP difference between these populations increased with age [6]. Homi et al. studying adolescents aged 16–18 years showed that the lower BP described in SCD compared to non SCD subjects could be accounted by weight differences [7]. In another small study, Rodgers et al. reported no significant differences in BP values prior to age 17 in a small cross-sectional cohort (*n* = 13) [8].

More recently, children with SCD have been found to have abnormal BP patterns [10–12]. Bodas et al., in a cross-sectional review, found that 8/48 (16.7%) children with SCD had abnormal BP (elevated BP or hypertension) [10]. The authors suggested that hypertension may be underdiagnosed in children with SCD using standard clinic based assessments. Utilizing 24-hour BP monitoring criteria [18], Shatat et al. reported that 18/52 (35%) of patients had previously unrecognized hypertension, 9/52 (17%) had elevated BP and 29/52 (56%) lacked the normal nocturnal dip in BP [11]. In addition, masked hypertension was found to be prevalent in children with SCD: while only 4/38 (10.3%) of these children were hypertensive based on clinic BP, 17/38 (43.6%) had ambulatory hypertension confirmed by 24-hour BP measurements [11].

### Strengths and limitations

This secondary analysis has notable limitations. First, the SIT trial BP measurement methods were not standardized across clinical sites without specification whether auscultatory or oscillometric measurement, although BPs were obtained by auscultatory methods in the 2017 CPG [9, 17]. Second, a median of 5 BP measurements was recorded in children with SCD over 3 years. This repeated measurement further supports our hypothesis because multiple measurements should have resulted in regression to the mean and lower BP values in SCD. However, they could also represent increasing BPs over time in SCD; therefore, the median of 5 measurements was higher.

Third, this analysis is premised on the assumption that BP measurements from the SIT trial represent the SCD population without comorbidities, to the extent that any comorbidities are not consequences of SCD, rather than general SCD population with all comorbidities.

Finally, the original trial report included little information on the characteristics of the SCD population, which included both children with and without silent strokes [16]. In addition, the trial only included children with HbSS or HbSβ°. Because the SIT trial is not representative of all children with SCD, our study may have limited external validity.

## Conclusions

Our comparative analysis compared an SCD population with reference BP values and demonstrated that systolic BP values are higher in children with SCD than in the general pediatric population, in contrast to previously reported. This finding is also consistent with the most recent literature showing abnormal BP patterns in the SCD pediatric population utilizing 24-hour BP monitoring devices. These are important steps for recognizing abnormal BP as a risk factor for cardio- and neurovascular events in SCD.

## Supplementary Information

**Additional file 1: Supplementary table 1**. Systolic and diastolic blood pressures for males and females ages 5-17 with and without Sickle Cell Disease.

## Data Availability

All data generated or analyzed during this study are included in this published article. The data and R code are available at: https://github.com/Misreporting/sickle-cell-disease-blood-pressure.

## Abbreviations

ABPM: Ambulatory blood pressure monitoring
BMI: Body mass index
BP: Blood pressure
CPG: Clinical Practice Guideline
SCD: Sickle cell disease
SIT: Silent Cerebral Infarct Multicenter Clinical Trial
