# Supplementary material for: Blood pressure in children with sickle cell disease is higher than in the general pediatric population
Source: BMC Pediatr. 2022 Sep 15;22:549. doi: 10.1186/s12887-022-03584-9 (PMC9476310; doi:10.1186/s12887-022-03584-9)
Supplement: Supplementary file 1 — Additional file 1: Supplementary table 1. Systolic and diastolic blood pressures for males and females ages 5-17 with and without Sickle Cell Disease. [file 12887_2022_3584_MOESM1_ESM.docx]

**Supplementary table 1**- Systolic and diastolic blood pressures for males and females ages 5-17 with and without Sickle Cell Disease

| **Males systolic** |  | **SCD** | **4th report 2004** | **CPG 2017** | **SCD** | **4th report 2004** | **CPG 2017** | **SCD** | **4th report 2004** | **CPG 2017** | **SCD** | **4th report 2004** | **CPG 2017** |
| --- | --- | --- | --- | --- | --- | --- | --- | --- | --- | --- | --- | --- | --- |
| **Age** | **Perc.** | **5th** | **5th** | **5th** | **50th** | **50th** | **50th** | **90th** | **90th** | **90th** | **95th** | **95th** | **95th** |
| **5** | **50th** | 102 | 90 | 91 | 105 | 95 | 94 | 107 | 98 | 96 | 107 | 98 | 96 |
|  | **90th** | 114 | 104 | 103 | 117 | 108 | 106 | 119 | 111 | 108 | 119 | 112 | 108 |
|  | **95th** | 121 | 108 | 107 | 124 | 112 | 109 | 126 | 115 | 111 | 126 | 116 | 112 |
| **6** | **50th** | 103 | 91 | 93 | 106 | 96 | 95 | 108 | 99 | 97 | 108 | 100 | 98 |
|  | **90th** | 115 | 105 | 105 | 118 | 110 | 107 | 120 | 113 | 110 | 120 | 113 | 110 |
|  | **95th** | 121 | 109 | 108 | 124 | 114 | 111 | 126 | 117 | 113 | 126 | 117 | 114 |
| **7** | **50th** | 104 | 92 | 94 | 107 | 97 | 97 | 109 | 100 | 98 | 109 | 101 | 99 |
|  | **90th** | 116 | 106 | 106 | 119 | 111 | 109 | 121 | 114 | 111 | 121 | 115 | 111 |
|  | **95th** | 121 | 110 | 110 | 124 | 115 | 112 | 126 | 118 | 115 | 126 | 119 | 116 |
| **8** | **50th** | 105 | 94 | 95 | 108 | 99 | 98 | 110 | 102 | 99 | 110 | 102 | 100 |
|  | **90th** | 117 | 107 | 107 | 120 | 112 | 110 | 122 | 115 | 112 | 122 | 116 | 112 |
|  | **95th** | 121 | 111 | 111 | 124 | 116 | 114 | 126 | 119 | 116 | 126 | 120 | 117 |
| **9** | **50th** | 106 | 95 | 96 | 109 | 100 | 99 | 111 | 103 | 101 | 112 | 104 | 101 |
|  | **90th** | 118 | 109 | 107 | 121 | 114 | 110 | 123 | 117 | 113 | 124 | 118 | 114 |
|  | **95th** | 121 | 113 | 112 | 124 | 118 | 115 | 126 | 121 | 118 | 127 | 121 | 119 |
| **10** | **50th** | 106 | 97 | 97 | 110 | 102 | 100 | 112 | 105 | 102 | 113 | 106 | 103 |
|  | **90th** | 119 | 111 | 108 | 122 | 115 | 112 | 124 | 119 | 115 | 125 | 119 | 116 |
|  | **95th** | 122 | 115 | 112 | 125 | 119 | 116 | 128 | 122 | 120 | 128 | 123 | 121 |
| **11** | **50th** | 107 | 99 | 99 | 110 | 104 | 102 | 114 | 107 | 104 | 114 | 107 | 106 |
|  | **90th** | 120 | 113 | 110 | 123 | 117 | 114 | 126 | 120 | 117 | 126 | 121 | 118 |
|  | **95th** | 124 | 117 | 114 | 127 | 121 | 118 | 129 | 124 | 123 | 130 | 125 | 124 |
| **12** | **50th** | 108 | 101 | 101 | 111 | 106 | 104 | 115 | 109 | 108 | 116 | 110 | 109 |
|  | **90th** | 121 | 115 | 113 | 124 | 120 | 117 | 127 | 123 | 121 | 128 | 123 | 122 |
|  | **95th** | 125 | 119 | 116 | 128 | 123 | 121 | 131 | 127 | 126 | 132 | 127 | 128 |
| **13** | **50th** | 109 | 104 | 103 | 112 | 108 | 108 | 116 | 111 | 111 | 117 | 112 | 112 |
|  | **90th** | 123 | 117 | 115 | 126 | 122 | 121 | 129 | 125 | 126 | 130 | 126 | 126 |
|  | **95th** | 127 | 121 | 119 | 130 | 126 | 125 | 133 | 129 | 130 | 134 | 130 | 131 |
| **14** | **50th** | 110 | 106 | 105 | 114 | 111 | 111 | 117 | 114 | 113 | 118 | 115 | 113 |
|  | **90th** | 124 | 120 | 119 | 128 | 125 | 126 | 131 | 128 | 128 | 132 | 128 | 129 |
|  | **95th** | 129 | 124 | 123 | 133 | 128 | 130 | 135 | 132 | 133 | 136 | 132 | 134 |
| **15** | **50th** | 111 | 109 | 108 | 115 | 113 | 113 | 118 | 117 | 114 | 119 | 117 | 114 |
|  | **90th** | 126 | 122 | 123 | 130 | 127 | 128 | 133 | 130 | 130 | 133 | 131 | 130 |
|  | **95th** | 131 | 126 | 127 | 135 | 131 | 132 | 138 | 134 | 135 | 138 | 135 | 135 |
| **16** | **50th** | 112 | 111 | 111 | 116 | 116 | 115 | 119 | 119 | 116 | 120 | 120 | 116 |
|  | **90th** | 128 | 125 | 126 | 132 | 130 | 129 | 135 | 133 | 131 | 135 | 134 | 132 |
|  | **95th** | 133 | 129 | 130 | 137 | 134 | 134 | 140 | 137 | 136 | 140 | 137 | 137 |
| **17** | **50th** | 113 | 114 | 114 | 117 | 118 | 117 | 120 | 121 | 118 | 121 | 122 | 118 |
|  | **90th** | 131 | 127 | 128 | 134 | 132 | 131 | 137 | 135 | 133 | 137 | 136 | 134 |
|  | **95th** | 136 | 131 | 132 | 139 | 136 | 135 | 142 | 139 | 138 | 142 | 140 | 138 |
|  | | | | | | | | | | | | | |
| **Males diastolic** |  | **SCD** | **4th report 2004** | **CPG 2017** | **SCD** | **4th report 2004** | **CPG 2017** | **SCD** | **4th report 2004** | **CPG 2017** | **SCD** | **4th report 2004** | **CPG 2017** |
| **Age** | **Perc** | **5th** | **5th** | **5th** | **50th** | **50th** | **50th** | **90th** | **90th** | **90th** | **95th** | **95th** | **95th** |
| **5** | **50th** | 59 | 50 | 51 | 60 | 53 | 53 | 60 | 55 | 55 | 60 | 55 | 55 |
|  | **90th** | 68 | 65 | 63 | 69 | 65 | 65 | 70 | 68 | 67 | 71 | 69 | 67 |
|  | **95th** | 72 | 69 | 66 | 74 | 69 | 69 | 76 | 72 | 70 | 76 | 74 | 71 |
| **6** | **50th** | 59 | 53 | 54 | 60 | 53 | 56 | 61 | 55 | 57 | 61 | 57 | 58 |
|  | **90th** | 68 | 68 | 66 | 70 | 68 | 68 | 71 | 70 | 69 | 71 | 72 | 69 |
|  | **95th** | 72 | 72 | 69 | 74 | 72 | 71 | 76 | 74 | 72 | 76 | 76 | 73 |
| **7** | **50th** | 60 | 55 | 56 | 60 | 55 | 58 | 61 | 57 | 59 | 61 | 59 | 59 |
|  | **90th** | 68 | 70 | 68 | 70 | 70 | 70 | 71 | 72 | 71 | 71 | 74 | 71 |
|  | **95th** | 72 | 74 | 71 | 74 | 74 | 73 | 76 | 76 | 74 | 76 | 78 | 74 |
| **8** | **50th** | 60 | 56 | 57 | 61 | 56 | 59 | 61 | 59 | 60 | 61 | 60 | 60 |
|  | **90th** | 69 | 71 | 69 | 70 | 71 | 71 | 72 | 73 | 72 | 72 | 75 | 73 |
|  | **95th** | 72 | 75 | 72 | 74 | 75 | 74 | 76 | 78 | 75 | 76 | 79 | 75 |
| **9** | **50th** | 60 | 57 | 57 | 61 | 57 | 60 | 62 | 60 | 62 | 62 | 61 | 62 |
|  | **90th** | 69 | 72 | 70 | 71 | 72 | 73 | 73 | 75 | 74 | 73 | 76 | 74 |
|  | **95th** | 73 | 76 | 74 | 75 | 76 | 76 | 77 | 79 | 77 | 77 | 81 | 77 |
| **10** | **50th** | 61 | 58 | 59 | 62 | 58 | 62 | 63 | 61 | 63 | 63 | 62 | 64 |
|  | **90th** | 71 | 73 | 72 | 72 | 73 | 74 | 74 | 75 | 75 | 74 | 77 | 76 |
|  | **95th** | 74 | 77 | 76 | 76 | 77 | 77 | 78 | 80 | 78 | 78 | 81 | 78 |
| **11** | **50th** | 62 | 59 | 61 | 62 | 59 | 63 | 63 | 61 | 63 | 63 | 63 | 63 |
|  | **90th** | 72 | 74 | 74 | 74 | 74 | 75 | 75 | 76 | 76 | 76 | 78 | 76 |
|  | **95th** | 75 | 78 | 77 | 77 | 78 | 78 | 79 | 80 | 78 | 79 | 82 | 78 |
| **12** | **50th** | 62 | 59 | 61 | 63 | 59 | 62 | 64 | 62 | 63 | 64 | 63 | 63 |
|  | **90th** | 72 | 74 | 75 | 74 | 74 | 75 | 76 | 76 | 76 | 77 | 78 | 76 |
|  | **95th** | 75 | 78 | 78 | 78 | 78 | 78 | 80 | 81 | 79 | 81 | 82 | 79 |
| **13** | **50th** | 62 | 60 | 61 | 63 | 60 | 62 | 64 | 62 | 64 | 64 | 64 | 65 |
|  | **90th** | 73 | 75 | 74 | 75 | 75 | 75 | 76 | 77 | 77 | 77 | 79 | 77 |
|  | **95th** | 76 | 79 | 78 | 78 | 79 | 78 | 80 | 81 | 81 | 81 | 83 | 81 |
| **14** | **50th** | 62 | 60 | 60 | 63 | 60 | 64 | 64 | 63 | 66 | 64 | 65 | 67 |
|  | **90th** | 72 | 75 | 74 | 74 | 75 | 77 | 76 | 78 | 79 | 77 | 79 | 80 |
|  | **95th** | 76 | 80 | 77 | 78 | 80 | 81 | 80 | 82 | 83 | 81 | 84 | 84 |
| **15** | **50th** | 62 | 61 | 61 | 63 | 61 | 65 | 64 | 64 | 67 | 64 | 66 | 68 |
|  | **90th** | 71 | 76 | 75 | 74 | 76 | 79 | 75 | 79 | 81 | 76 | 80 | 81 |
|  | **95th** | 75 | 81 | 78 | 78 | 81 | 83 | 80 | 83 | 85 | 80 | 85 | 85 |
| **16** | **50th** | 61 | 63 | 63 | 63 | 63 | 67 | 63 | 65 | 69 | 64 | 67 | 69 |
|  | **90th** | 70 | 78 | 77 | 73 | 78 | 80 | 74 | 80 | 82 | 75 | 82 | 82 |
|  | **95th** | 75 | 82 | 80 | 77 | 82 | 84 | 79 | 84 | 86 | 80 | 86 | 86 |
| **17** | **50th** | 61 | 65 | 65 | 62 | 65 | 68 | 63 | 67 | 70 | 63 | 69 | 70 |
|  | **90th** | 70 | 80 | 78 | 72 | 80 | 81 | 73 | 82 | 82 | 74 | 84 | 83 |
|  | **95th** | 74 | 84 | 81 | 77 | 84 | 85 | 79 | 87 | 86 | 79 | 88 | 87 |
|  | | | | | | | | | | | | | |
| **Females systolic** |  | **SCD** | **4th report 2004** | **CPG 2017** | **SCD** | **4th report 2004** | **CPG 2017** | **SCD** | **4th report 2004** | **CPG 2017** | **SCD** | **4th report 2004** | **CPG 2017** |
| **Age** | **Perc** | **5th** | **5th** | **5th** | **50th** | **50th** | **50th** | **90th** | **90th** | **90th** | **95th** | **95th** | **95th** |
| **5** | **50th** | 100 | 89 | 90 | 103 | 93 | 93 | 105 | 95 | 95 | 105 | 96 | 96 |
|  | **90th** | 115 | 103 | 104 | 118 | 106 | 107 | 120 | 109 | 109 | 120 | 109 | 110 |
|  | **95th** | 118 | 107 | 108 | 121 | 110 | 110 | 122 | 112 | 112 | 123 | 113 | 113 |
| **6** | **50th** | 101 | 91 | 92 | 104 | 94 | 94 | 106 | 97 | 97 | 107 | 98 | 97 |
|  | **90th** | 116 | 104 | 105 | 119 | 108 | 108 | 120 | 110 | 110 | 121 | 111 | 111 |
|  | **95th** | 119 | 108 | 109 | 122 | 111 | 111 | 123 | 114 | 113 | 124 | 115 | 114 |
| **7** | **50th** | 103 | 93 | 92 | 106 | 96 | 95 | 108 | 99 | 98 | 109 | 99 | 99 |
|  | **90th** | 117 | 106 | 106 | 119 | 109 | 109 | 121 | 112 | 111 | 122 | 113 | 112 |
|  | **95th** | 120 | 110 | 109 | 123 | 113 | 112 | 124 | 116 | 114 | 125 | 116 | 115 |
| **8** | **50th** | 105 | 95 | 93 | 108 | 98 | 97 | 110 | 100 | 99 | 111 | 101 | 100 |
|  | **90th** | 118 | 108 | 107 | 120 | 111 | 110 | 122 | 114 | 112 | 123 | 114 | 113 |
|  | **95th** | 121 | 112 | 110 | 124 | 115 | 113 | 126 | 118 | 116 | 126 | 118 | 117 |
| **9** | **50th** | 106 | 96 | 95 | 109 | 100 | 98 | 112 | 102 | 100 | 112 | 103 | 101 |
|  | **90th** | 119 | 110 | 108 | 121 | 113 | 111 | 124 | 116 | 113 | 124 | 116 | 114 |
|  | **95th** | 123 | 114 | 112 | 125 | 117 | 114 | 127 | 119 | 117 | 128 | 120 | 118 |
| **10** | **50th** | 107 | 98 | 96 | 110 | 102 | 99 | 113 | 104 | 102 | 113 | 105 | 103 |
|  | **90th** | 120 | 112 | 109 | 123 | 115 | 112 | 125 | 118 | 115 | 126 | 118 | 116 |
|  | **95th** | 124 | 116 | 113 | 127 | 119 | 116 | 129 | 121 | 119 | 130 | 122 | 120 |
| **11** | **50th** | 107 | 100 | 98 | 111 | 103 | 102 | 114 | 106 | 105 | 115 | 107 | 106 |
|  | **90th** | 121 | 114 | 111 | 124 | 117 | 114 | 127 | 119 | 118 | 127 | 120 | 120 |
|  | **95th** | 126 | 118 | 115 | 129 | 121 | 118 | 131 | 123 | 123 | 132 | 124 | 124 |
| **12** | **50th** | 108 | 102 | 102 | 112 | 105 | 105 | 115 | 108 | 108 | 116 | 109 | 108 |
|  | **90th** | 122 | 116 | 114 | 125 | 119 | 118 | 128 | 121 | 122 | 128 | 122 | 122 |
|  | **95th** | 127 | 119 | 118 | 130 | 123 | 122 | 133 | 125 | 125 | 134 | 126 | 126 |
| **13** | **50th** | 109 | 104 | 104 | 113 | 107 | 107 | 116 | 110 | 108 | 117 | 110 | 109 |
|  | **90th** | 122 | 117 | 116 | 126 | 121 | 121 | 129 | 123 | 123 | 129 | 124 | 123 |
|  | **95th** | 127 | 121 | 121 | 131 | 124 | 124 | 134 | 127 | 126 | 134 | 128 | 127 |
| **14** | **50th** | 109 | 106 | 105 | 113 | 109 | 108 | 117 | 111 | 109 | 118 | 112 | 109 |
|  | **90th** | 123 | 119 | 118 | 126 | 122 | 122 | 129 | 125 | 123 | 130 | 125 | 123 |
|  | **95th** | 128 | 123 | 123 | 131 | 126 | 125 | 134 | 129 | 127 | 135 | 129 | 127 |
| **15** | **50th** | 110 | 107 | 105 | 114 | 110 | 108 | 117 | 113 | 109 | 119 | 113 | 109 |
|  | **90th** | 123 | 120 | 118 | 127 | 123 | 122 | 129 | 126 | 123 | 130 | 127 | 124 |
|  | **95th** | 128 | 124 | 124 | 131 | 127 | 126 | 134 | 130 | 127 | 135 | 131 | 128 |
| **16** | **50th** | 112 | 108 | 106 | 115 | 111 | 109 | 118 | 114 | 110 | 120 | 114 | 110 |
|  | **90th** | 124 | 121 | 119 | 127 | 124 | 123 | 129 | 127 | 124 | 131 | 128 | 124 |
|  | **95th** | 127 | 125 | 124 | 130 | 128 | 127 | 133 | 131 | 128 | 134 | 133 | 128 |
| **17** | **50th** | 113 | 108 | 107 | 116 | 111 | 110 | 119 | 114 | 110 | 120 | 115 | 111 |
|  | **90th** | 124 | 122 | 120 | 127 | 125 | 124 | 130 | 127 | 125 | 131 | 128 | 125 |
|  | **95th** | 127 | 125 | 125 | 130 | 129 | 127 | 133 | 131 | 128 | 134 | 132 | 128 |
|  | | | | | | | | | | | | | |
| **Females diastolic** |  | **SCD** | **4th report 2004** | **CPG 2017** | **SCD** | **4th report 2004** | **CPG 2017** | **SCD** | **4th report 2004** | **CPG 2017** | **SCD** | **4th report 2004** | **CPG 2017** |
| **Age** | **Perc** | **5th** | **5th** | **5th** | **50th** | **50th** | **50th** | **90th** | **90th** | **90th** | **95th** | **95th** | **95th** |
| **5** | **50th** | 60 | 52 | 52 | 61 | 54 | 55 | 61 | 55 | 57 | 61 | 56 | 57 |
|  | **90th** | 70 | 66 | 64 | 71 | 68 | 67 | 72 | 69 | 69 | 73 | 70 | 70 |
|  | **95th** | 72 | 70 | 68 | 74 | 72 | 71 | 75 | 73 | 73 | 76 | 74 | 73 |
| **6** | **50th** | 60 | 54 | 54 | 61 | 56 | 56 | 61 | 57 | 58 | 61 | 58 | 59 |
|  | **90th** | 69 | 68 | 67 | 71 | 70 | 69 | 72 | 71 | 71 | 72 | 72 | 71 |
|  | **95th** | 72 | 72 | 70 | 74 | 74 | 72 | 75 | 75 | 74 | 76 | 76 | 74 |
| **7** | **50th** | 60 | 55 | 55 | 61 | 57 | 57 | 61 | 58 | 59 | 62 | 59 | 60 |
|  | **90th** | 69 | 69 | 68 | 70 | 71 | 70 | 72 | 72 | 72 | 72 | 73 | 72 |
|  | **95th** | 72 | 73 | 72 | 74 | 75 | 73 | 75 | 76 | 74 | 76 | 77 | 75 |
| **8** | **50th** | 60 | 57 | 56 | 61 | 58 | 59 | 62 | 60 | 61 | 62 | 60 | 61 |
|  | **90th** | 69 | 71 | 69 | 70 | 72 | 72 | 71 | 74 | 73 | 72 | 74 | 73 |
|  | **95th** | 72 | 75 | 72 | 74 | 76 | 74 | 75 | 78 | 75 | 76 | 78 | 75 |
| **9** | **50th** | 61 | 58 | 57 | 61 | 59 | 60 | 62 | 61 | 61 | 62 | 61 | 61 |
|  | **90th** | 69 | 72 | 71 | 70 | 73 | 73 | 72 | 75 | 73 | 72 | 75 | 73 |
|  | **95th** | 73 | 76 | 74 | 74 | 77 | 75 | 76 | 79 | 75 | 76 | 79 | 75 |
| **10** | **50th** | 61 | 59 | 58 | 62 | 60 | 60 | 62 | 62 | 61 | 63 | 62 | 62 |
|  | **90th** | 69 | 73 | 72 | 71 | 74 | 73 | 73 | 76 | 73 | 73 | 76 | 73 |
|  | **95th** | 73 | 77 | 75 | 75 | 78 | 76 | 77 | 80 | 76 | 78 | 80 | 76 |
| **11** | **50th** | 61 | 60 | 60 | 62 | 61 | 61 | 63 | 63 | 63 | 63 | 63 | 64 |
|  | **90th** | 70 | 74 | 74 | 72 | 75 | 74 | 74 | 77 | 75 | 74 | 77 | 75 |
|  | **95th** | 74 | 78 | 76 | 77 | 79 | 77 | 78 | 81 | 77 | 79 | 81 | 77 |
| **12** | **50th** | 62 | 61 | 61 | 63 | 62 | 62 | 64 | 64 | 65 | 64 | 64 | 65 |
|  | **90th** | 71 | 75 | 75 | 73 | 76 | 75 | 75 | 57 | 76 | 75 | 78 | 76 |
|  | **95th** | 75 | 79 | 78 | 78 | 80 | 78 | 80 | 82 | 79 | 80 | 82 | 79 |
| **13** | **50th** | 62 | 62 | 62 | 63 | 63 | 64 | 64 | 65 | 65 | 64 | 65 | 66 |
|  | **90th** | 71 | 76 | 75 | 73 | 77 | 76 | 75 | 79 | 76 | 76 | 79 | 76 |
|  | **95th** | 76 | 80 | 79 | 78 | 81 | 79 | 80 | 83 | 80 | 81 | 83 | 81 |
| **14** | **50th** | 63 | 63 | 63 | 64 | 64 | 65 | 64 | 66 | 66 | 65 | 66 | 66 |
|  | **90th** | 72 | 77 | 76 | 74 | 78 | 76 | 75 | 80 | 77 | 76 | 80 | 77 |
|  | **95th** | 76 | 81 | 80 | 78 | 82 | 80 | 80 | 84 | 81 | 81 | 84 | 82 |
| **15** | **50th** | 63 | 64 | 64 | 64 | 65 | 65 | 65 | 67 | 67 | 65 | 67 | 67 |
|  | **90th** | 72 | 78 | 76 | 74 | 79 | 77 | 75 | 81 | 78 | 76 | 81 | 78 |
|  | **95th** | 76 | 82 | 80 | 78 | 83 | 81 | 80 | 85 | 82 | 81 | 85 | 82 |
| **16** | **50th** | 63 | 64 | 64 | 64 | 66 | 66 | 65 | 67 | 67 | 66 | 68 | 67 |
|  | **90th** | 72 | 78 | 76 | 74 | 80 | 77 | 75 | 81 | 78 | 76 | 82 | 78 |
|  | **95th** | 76 | 82 | 80 | 78 | 84 | 81 | 80 | 85 | 82 | 81 | 86 | 82 |
| **17** | **50th** | 64 | 64 | 64 | 65 | 66 | 66 | 66 | 67 | 66 | 66 | 68 | 67 |
|  | **90th** | 72 | 78 | 76 | 74 | 80 | 77 | 75 | 81 | 78 | 76 | 82 | 78 |
|  | **95th** | 76 | 82 | 80 | 78 | 84 | 81 | 80 | 85 | 82 | 81 | 86 | 82 |
